# Successful Mechanical Thrombectomy for Basilar Artery Occlusion in a Seven-Year-Old Male

**DOI:** 10.7759/cureus.13950

**Published:** 2021-03-17

**Authors:** Nasar Ali, Mustafa Al-Chalabi, Hisham Salahuddin

**Affiliations:** 1 Department of Neurology, University of Toledo, Toledo, USA

**Keywords:** mechanical thrombectomy in pediatrics, pediatric stroke, basilar artery occlusion, vertebral artery dissection, trampoline

## Abstract

Acute arterial strokes in children are rare but can potentially cause lasting and often permanent neurological deficits. Mechanical thrombectomy has a well-established efficacy and safety profile in adult stroke management, but in the pediatric population, it is yet to be proven efficacious and safe. We present a case of a seven-year-old male who presented with multiple episodes of generalized tonic-clonic seizures after sustaining a neck injury by falling from a trampoline. National Institutes of Health (NIH) on presentation was 21. Neurological exam revealed dilated nonreactive pupils, dysconjugate gaze, severe dysarthria, bilateral ptosis, and movement of upper and lower extremities only to noxious stimuli. Magnetic resonance imaging (MRI) of brain without contrast revealed infarcted areas in the left pons, midbrain, and cerebellar regions. Computed tomographic angiogram (CTA) of head demonstrated left vertebral artery dissection with associated complete occlusion of the distal basilar artery. Successful recanalization was achieved with mechanical thrombectomy six hours after presentation. Mechanical thrombectomy treatment resulted in a significant neurological recovery with NIH of 1. This case supports the growing evidence of the efficacy and safety of mechanical thrombectomy in children.

## Introduction

Although the morality of arterial ischemic stroke in children is less than that of adult arterial ischemic stroke, it still can cause a significant morbidity. Neurological deficits in children who suffer from strokes can be debilitating [1]. A permanent motor deficit occurs in 50% of neonates and in 65% of children older than one month as a result of stroke [1]. The occurrence of stroke in children is quite rare with an approximation of two to eight per 100,000 children per year [1]. Acute ischemic stroke (AIS) represents nearly half of the strokes in children [1]. The most common cause of AIS in children is cardiac diseases [1]. The mortality rate in children with AIS is 3%-6%, and those who survive have a recurrence AIS rate of 25% [2]. Seventy percent of the children that have survived an AIS are left with life-long disability causing social and personal constraints [2]. Mechanical thrombectomy revolutionized stroke care in adults with well-established guidelines and well-studied results from numerous randomized clinical trials [2]. However, its role is unknown in the pediatric world due to the lack of randomized trials that address its efficacy and safety in children. Nevertheless, there is a growing evidence of safety and efficacy in performing mechanical thrombectomy in children, which stems mainly from case reports and retrospective studies. Here we present a pediatric case with good neurological outcomes after mechanical thrombectomy with the use of Sophia 6 French catheter (MicroVention, Inc., California, USA).

## Case presentation

A seven-year-old male in the United States without significant past medical history presented with three episodes of generalized tonic-clonic seizures. These seizures occurred consecutively without regaining consciousness. A day prior, he fell off a trampoline where he sustained neck trauma followed by mild neck pain with associated nausea and vomiting. Since there was no loss of consciousness or any focal neurological trauma, the patient was not taken to the emergency department (ED) on initial trauma. On arrival at the ED the next day, he was noted with right gaze deviation and was unresponsive. Computed tomography (CT) of the head without contrast did not reveal any acute pathology. Routine labs were within normal limits including complete blood count with differential, comprehensive metabolic panel, prothrombin time/international randomized ratio, salicylate, and acetaminophen; alcohol level was less than 0.01, and urine drug screen was negative. He was loaded with one gram of levetiracetam with a maintenance of 180 mg twice daily. For airway protection, he was intubated and was admitted to the pediatric Intensive Care Unit (ICU) for further care and management.

**Figure 1:**
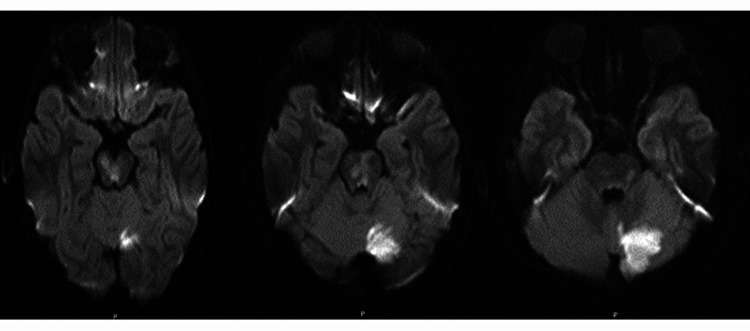
DWI sequence with ischemic changes in the midbrain, pons, and left cerebellum. The hyperintensities noted in the frontal lobes and temporal lobes are susceptibility artifacts. DWI, Diffusion-weighted imaging.

**Figure 2:**
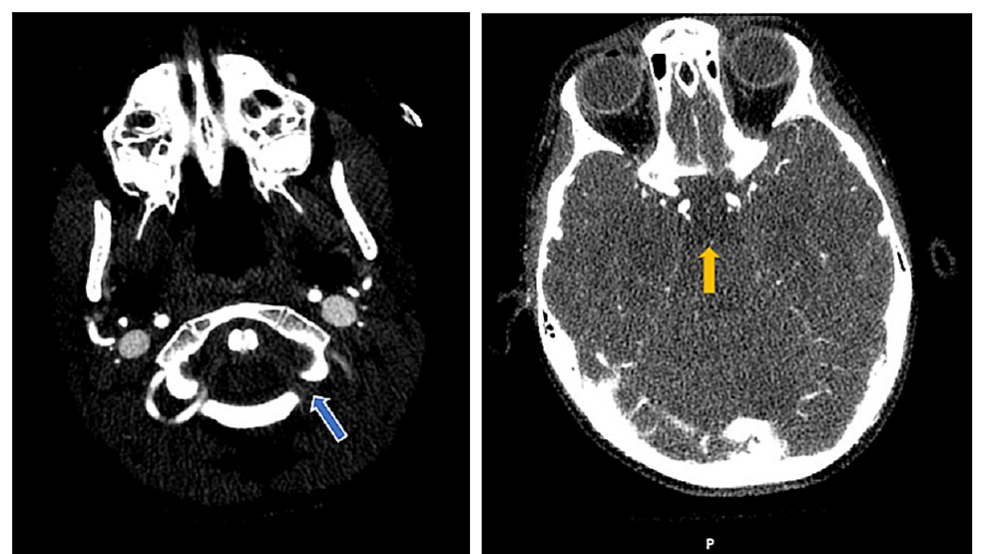
CTA showing the left vertebral artery dissection with associated complete occlusion of left V3 segment of VA (blue arrow) and distal basilar artery thrombosis (yellow arrow). CTA, Computed tomographic angiogram; VA, vertebral artery.

**Figure 3:**
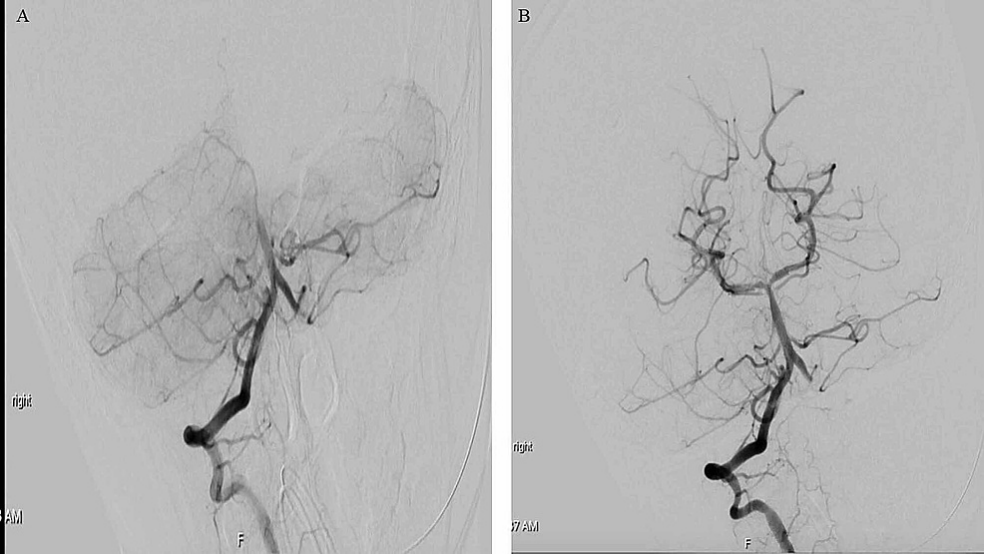
(A) Pre-thrombectomy four-vessel angiogram and (B) post-thrombectomy four-vessel angiogram.

Seizure workup included magnetic resonance imaging (MRI) of brain without contrast, which revealed areas of infarction in the left pons, midbrain, and cerebellar regions (Figure 1). Due to these findings, computed tomography angiograms (CTA) of the head and neck were obtained revealing left vertebral artery dissection with associated complete occlusion of the left V3 segment of vertebral artery (VA) and distal basilar artery thrombosis (Figure 2). The stroke intervention team was notified of these findings, and the patient was taken for a successful emergent mechanical thrombectomy with Sophia 6 French catheter (Figure 3) after a repeat rapid brain imaging protocol consisting of diffusion-weighted imaging (DWI) and apparent diffusion coefficient (ADC) sequence did not reveal the expansion of ischemic changes compared to prior MRI. At the end of the procedure, a successful thrombolysis in cerebral infarction 3 (TICI3) recanalization was achieved. Of note, the mechanical thrombectomy was performed 6.5 hours after the time of presentation to the ED.

Prior to performing mechanical thrombectomy, the National Institutes of Health Stroke Scale (NIHSS) was 21. However, the patient was intubated and sedated. General neurological exam revealed the following: intact brainstem reflexes, dysconjugate gaze, withdrawal to noxious stimuli in the right side, and extensor response with noxious stimuli in the left side.

He was successfully extubated several hours after undergoing mechanical thrombectomy. Post extubation and mechanical thrombectomy neurological exam showed ability to follow commands, complete resolution of dysarthria within three days, persistent bilateral ptosis, 4 mm reactive pupils bilaterally, persistent dysconjugate gaze secondary to left sixth nerve palsy, and intact movement of all extremities except for left upper extremity ataxia. On the four-month follow-up visit, the patient had good neurological recovery with NIHSS of 1 for left upper extremity ataxia. He was discharged on levetiracetam for seizure prophylaxis for a period of six months and was slowly weaned off. Additionally, he was discharged on aspirin, which was also discontinued at six months.

## Discussion

Our seven-year-old male patient presented with status epilepticus as a result of brainstem and cerebellar strokes. The mechanism of the stroke in our patient was artery-to-artery embolism caused by left VA dissection with associated distal basilar artery thrombosis. The dissection was caused by a fall from a trampoline. MRI of the brain without contrast revealed infarcted areas in the left pons, midbrain, and cerebellar regions. The patient underwent mechanical thrombectomy using Sophia 6 French catheter resulting in promising neurological outcome at six-month follow-up. The persistent deficits at the six-month follow-up period included left upper extremity ataxia and difficulty with upward gaze.

The most common presenting sign of ischemic stroke in children is a focal neurological deficit. However, recognizing ischemic stroke symptoms in children can be difficult considering it is uncommon in this population and is likely masqueraded by multiple mimics. Stroke mimics include seizure, migraine, demyelinating disease, encephalitis, mass lesion, posterior reversible encephalopathy syndrome (PRES), and functional neurological disorder [3]. Cognitive dysfunctions such as language deficits are present in 60% of children following a stroke [4]. Therefore, early recognition of stroke symptoms as well as having a robust and quick multidisciplinary approach analogous to that in place for adults are detrimental in stroke management in children. A seizure can be an early sign of acute stroke in children, and it should prompt a stroke investigatory workup [3]. Recurrent focal seizures, usually focal in neonates, can be a manifestation of middle cerebral artery stroke in this population. Hemiplegia, developmental delay, and epilepsy are also among the sequelae of ischemic stroke in infants, but they are more noticeable in four to six months of age [4].

Cardioembolic phenomenon constitutes the majority of arterial ischemic stroke etiologies in children. Around 30% of children with ischemic stroke are found to have acquired or congenital cardiac diseases [5]. Cerebral arteriopathy is associated with as much as 50% of arterial ischemic stroke cases in children [6]. Cerebral arteriopathy includes focal cerebral arteriopathy, moyamoya, dissection, vasculitis, sickle cell arteriopathy, and post varicella angiopathy. Focal cerebral arteriopathy, which is a stenosis in the large- to medium-sized arteries, represents one-fourth of the cerebral arteriopathies in children. Chronic inflammation or infection is implicated in the pathogenesis of focal cerebral arteriopathy [7]. Up to 20% of ischemic stroke in children is caused by cervicocephalic dissection, and it constitutes 50% of posterior childhood ischemic strokes. Cervicocephalic dissection can happen after trauma or spontaneously. Patients with concurrent connective tissue disorders are more prone to have traumatic dissection [7-9]. Hematological disorders that are associated with ischemic stroke in children include sickle cell disease, iron deficiency anemia, thrombocytosis, and malignancy. Hypercoagulable disorders that are associated with ischemic stroke in children include homocysteinemia, anticardiolipin antibodies, elevated lipoprotein (a), protein C deficiency, protein S deficiency, factor V Leiden, and factor II (prothrombin mutation G20210A). Lastly, systemic lupus erythematosus, rheumatoid arthritis, and systemic vasculitis are among the systemic disorders that are associated with ischemic stroke in children [7-10].

During the acute phase of AIS, mechanical thrombectomy can be just as safe and effective as giving tissue plasminogen activator (tPA) [11]. Bhogal et al. performed a retrospective study of five cases, ages ranging from seven to 17 years old that showed the efficacy of mechanical thrombectomy in the pediatric population [11]. Four of the cases involved the anterior circulation (left M1 or M2 segment), and one affected the posterior circulation (left VA). [11] In all of the five cases, the patients had good outcomes with the same devices used in the adult population [11]. The devices include pREset and pREset Lite stent retrievers (phenox GmbH, Bochum, Germany), Prowler Select Plus microcatheter (Codman Neuro, Massachusetts, USA), 5Fr ReFlex catheter (Covidien, Irvine, California), and Alligator device (Medtronic, Dublin, Ireland) [11]. Furthermore, a recent retrospective, multicenter cohort study by Sporns et al. showed that mechanical thrombectomy in children had a similar safety profile compared to adult trials [12]. The study was based on 27 centers in Europe and the United States, which focused on children under the age of 18 diagnosed with AIS who underwent endovascular recanalization [12]. Their findings suggested that endovascular thrombectomy in the pediatric population with large-vessel occlusion did not differ from the safety profile in randomized clinical trials for adults since most treated children had favorable neurological outcomes and low intraoperative complications [12].

Mechanical thrombectomy has not only been shown to be safe and effective, but a recent retrospective study conducted by Cobb et al. concluded that mechanical thrombectomy was superior in recanalization, clinical, and radiographic outcomes [13]. The rate of complications was also reduced with the use of mechanical thrombectomy [13]. The study included 68 pediatric patients whose age range was 1.8-18 years old, experiencing both posterior and anterior AIS, and were treated with either intravenous thrombolytics or mechanical thrombectomy with and without thrombolytics [13]. Furthermore, the study demonstrated high standards of safety and practicality of mechanical thrombectomy in children with large-vessel occlusions if performed by experienced neurointerventionalist using FDA-approved devices [13]. Parallel safety results of endovascular therapy in children less than 18-year-old and adults treated according to randomized-controlled trials were shown in a retrospective cohort study called “The Save ChildS Study” [14]. It revealed that the rate of recanalization, complications, and clinical outcomes did not correlate with stent retriever sizes used in endovascular thrombectomy, suggesting that endovascular therapy (EVT) should be considered in children presenting with large-vessel occlusion stroke irrespective of technical preference and devices availability [14].

## Conclusions

Although guidelines for mechanical thrombectomy in children with ischemic stroke are lacking, our case adds to the growing evidence that mechanical thrombectomy with catheters designed for adult patients may be safe and efficacious in the pediatric population.
